# Forecasting stock prices changes using long-short term memory neural network with symbolic genetic programming

**DOI:** 10.1038/s41598-023-50783-0

**Published:** 2024-01-03

**Authors:** Qi Li, Norshaliza Kamaruddin, Siti Sophiayati Yuhaniz, Hamdan Amer Ali Al-Jaifi

**Affiliations:** 1 Razak Faculty of Technology and Informatics, UTM Malaysia, Kuala Lumpur, Malaysia; 2 School of Accounting and Finance, Faculty of Business and Law, Taylor’s University, 47500 Subang Jaya, Selangor Malaysia

**Keywords:** Computer science, Statistics

## Abstract

This study introduces an augmented Long-Short Term Memory (LSTM) neural network architecture, integrating Symbolic Genetic Programming (SGP), with the objective of forecasting cross-sectional price returns across a comprehensive dataset comprising 4500 listed stocks in the Chinese market over the period from 2014 to 2022. Using the S&P Alpha Pool Dataset for China as basic input, this architecture incorporates data augmentation and feature extraction techniques. The result of this study demonstrates significant improvements in Rank Information coefficient (Rank IC) and IC information ratio (ICIR) by 1128% and 5360% respectively when it is applied to fundamental indicators. For technical indicators, the hybrid model achieves a 206% increase in Rank IC and an impressive surge of 2752% in ICIR. Furthermore, the proposed hybrid SGP-LSTM model outperforms major Chinese stock indexes, generating average annualized excess returns of 31.00%, 24.48%, and 16.38% compared to the CSI 300 index, CSI 500 index, and the average portfolio, respectively. These findings highlight the effectiveness of SGP-LSTM model in improving the accuracy of cross-sectional stock return predictions and provide valuable insights for fund managers, traders, and financial analysts.

## Introduction

Predicting the Stock return is a challenging endeavour, given the nonlinear nature of the stock market and the different approaches to predict the stock change. Though, advancements in artificial intelligence and other superior models have been used to increase forecasting accuracy, the prediction accuracy rate is still an unresolved issues^1^.

Enormous amount of attention in the empirical asset pricing literature has been directed to answer the questions of what drives the stock prices and what input features play major role in generating accurate results. In early years, Fama proposed in a weak-form market, people can make abnormal returns by mastering fundamental information, such as financial statements^2^. However, many scholars doubt the financial ratios do not consistently outperform the historical average benchmark forecast out of sample^3^. In addition other researchers started with the price trend itself, using technical indicators and found that technical indicators were efficient in predicting the market in the past^3^.

Thomas Fischer's 2018 study in the US market, utilizing the LSTM model, stands as one of the most referenced papers in cross-sectional stock selection. It unveiled a 51.4% accuracy rate between 1992 and 2015. However, the assessment of alpha between 2010 and 2015 showed a stagnant cumulative alpha of zero, highlighting limitations in this strategy during that period^4^. A subsequent parallel study by Ghosh et al. expanded input variables from one to three features but omitted classification accuracy. Notably, it demonstrated significant alpha improvements with a positive trajectory from 2010 to 2015, which unfortunately turned negative from 2016 to 2019^5^.

The persistence of challenges in maintaining consistent alpha generation seems to stem from limitations in input variables. Hanauer et al. addressed this issue by integrating fundamental indicators into their machine learning (ML) models, resulting in an average risk-adjusted alpha of approximately 6% for European stocks^6^. In contrast, Liu et al. study focusing on China's stock market employed a Deep Neural Network (DNN) incorporating 36 price-related trend features and 5 fundamental factors. Despite initially achieving a validation accuracy of 55.46%, the subsequent inclusion of trend-related features led to a decrease in accuracy, reaching 49.79% and falling short of the established 50% benchmark^7^.

As observed in the previous paragraph, advancements in input sources have significantly bolstered the accuracy and alpha performance within DNN models. This progress is especially notable in their proficiency for pattern recognition and predicting price changes, resulting in substantial enhancements in alpha generation. However, despite these advancements, several drawbacks persist, primarily centred around challenges in data integration and feature engineering. Multiple data sources exist, including technical and fundamental indicators, yet a comprehensive framework for their cohesive integration remains absent.

From the array of fundamental and technical indicators discussed earlier, the initial selection of features often involves manual intervention. This process heavily leans on existing domain expertise to guide and determine which features are chosen for inclusion in the analysis^8,9^. The ascent of smart beta investing has significantly reshaped the financial domain. Over the last decade, the surge in smart beta funds has been remarkable. In the past, the market exhibited a higher prevalence of discernible anomalies or 'alpha features'. However, the adoption of smart beta strategies based on existing alpha formulas by more funds has led to a decline in alpha effects due to increased capital flow. Consequently, even seasoned experts in the field face mounting challenges in identifying distinctive features. The pursuit of formula-driven, linear, and easily explicable features—vital elements in expert-driven extraction—is becoming less effective. This has spurred the emergence of AI-based feature engineering methods.

This paper aims to elevate the accuracy of cross-sectional stock return prediction and augment the average risk-adjusted return ('alpha') within the DNN framework. It builds upon Thomas Fischer's LSTM model by integrating additional input sources and proposing a novel feature engineering method involving symbolic genetic programming (SGP). This approach aims to address feature engineering limitations, enriching both fundamental and technical features. Furthermore, tailored LSTM models are crafted to suit the distinctive attributes of the dataset. Consequently, significant enhancements in accuracy, precision, and recall rates are observed, surpassing the performance of both Thomas Fischer's and Ghosh's LSTM models. Additionally, our method notably amplifies the Rank Information Coefficient (IC) and the Information Ratio of IC (ICIR), resulting in a substantial improvement in alpha compared to the benchmarks set within the frameworks of Fischer and Ghosh.

Moreover, we aim to synthesize the findings of our study into a simple and rule-based strategy for a complete active index fund strategy for selecting winning and losing stocks, compared with the benchmark. Our hybrid model exhibits superior performance compared to the CSI 300 and CSI 500 indexes. Notably, our strategy consistently outperforms these indexes by an average of 31% and 24.48% per year, respectively. Additionally, it surpasses the average returns of the entire market by 17.38% annually. We also calculate the information ratio of the strategy, and it is found that it is 2.49, and this further highlighting its effectiveness.

The remaining sections of this paper are organized as follows: Section "Related works" will cover related works, including existing DNN models and their combinations with Genetic Algorithms. In Section "The proposed deep neural network", we provide an in-depth discussion of the methodology, including enhanced SGP for new features, the proposed architecture of the Symbolic Genetic Programming (SGP-DNN model), input data descriptions, forecasting horizon, segmentation predictions method and the trading strategy setting. Section "Experiments with five classical DNN frameworks for comparison" will focus on the experiments. Section "Conclusion" will conclude the paper.

## Related works

The earliest study on applying machine learning in the stock domain can be traced back to 2006, where an accurate event weighting method and an automated event extraction system were presented^10^. However, there are several limitations to machine learning models. The challenges come from the employed dataset. Traditional machine learning models are best suited for small or medium-sized datasets and have limitations in processing high-dimensional datasets. They are prone to encountering the curse of dimensionality, especially for big or massive datasets, such as high-frequency or unstructured data^11^.

Comparing with machine learning algorithms, the Deep Neural Networks (DNNs) have significant advantages when it comes to handling large sets of time series data. LSTM is the most used model and advantageous over the conventional RNN due to the reason that it overcomes the problems of gradient vanishing or exploding. In 2015, Chen et al. built an LSTM-based model for the China stock market^12^. The most referenced paper for LSTM in the application in finance data was done by Thomas Fischer and Benedikt Kraus. They were the first to deploy the LSTM network on large-scale financial time series data and explained the source of the black box, which is high volatility, below-mean momentum, and extremal directional movement^4^. Following Fisher's work, four primary variants or supplementary approaches emerged as extensions to the single LSTM model: data decomposition, data dimension reduction, data augmentation techniques and Genetic Algorithm (GA) combination techniques.

Primarily, in the realm of data decompositions, traditional methods such as wavelet de-noising has been employed to stock index prediction since 2019^13,14^. The utilization of state-of-the-art techniques like Empirical Mode Decomposition (EMD) and Complex Empirical Mode Decomposition (CEEMD) has been prominent. This trend has notably continued since 2020. EMD and CEEMD have been notably applied to indices like the SP500, Dow Jones, HSI, DAX, SSE, and Nikkei. These methods break down the data into 6 to 8 frequency components, which are subsequently fed either individually or collectively (alongside residuals) into different Convolutional Neural Networks (CNNs). The output from these CNNs is then directed to LSTM components, or in some cases, directly to individual LSTM components. This complex pipeline is designed for the purpose of forecasting index price changes^15,16^.

Secondly data dimension reduction techniques have also been used with LSTM, numerous scholars have integrated Principal Component Analysis (PCA) with DNN models to achieve dimension reduction. Yong’an Zhang introduced the CEEMD-PCA-LSTM model for time series prediction. Preceding the LSTM model, input sources undergo processing by a PCA model to condense dimensions, thereby extracting abstract and advanced features. This process not only enhances computational efficiency but also contributes to improved predictive capabilities^17^. By 2023, even transformer models with fused multi-source features have been proposed, leveraging the ITD (intrinsic time-scale decomposition) method to manage feature dimensions effectively^18^.

Third supplementary approach is data augmentation. Fisher’s attempt of LSTM is single LSTM module, and the attributes of overfitting was challenged by other scholars due to the limited availability of data points. Yujin presented a novel data augmentation approach to avoid the overfitting and propose ModAugNet Framework including two modules, one is overfitting prevention LSTM module, and another is prediction LSTM module. The number of data point has been increased by 252 times^19^. We could also find the Phase Space Reconstruction (PSR) method^13^ or feature expansion method^20^ for data augmentation.

Finally, the combination of Genetic Algorithm (GA) and Deep Neural Network (DNN) or other Machine Learning models has been utilized by many researchers to improve prediction accuracy. For the application of GA in conjunction with Deep Neural Networks (DNNs), two main applications can be observed: hyperparameter tuning and feature selection.

Hyperparameter tuning is a crucial aspect that needs to be addressed in the optimization process, including parameters setting such as the number of layers, nodes per layer, and number of time lags. GA is frequently employed to search for optimal hyperparameters for DNN. In 2018, Chung and Shin employed GA to identify the optimal number of time lags and LSTM units for hidden layers in stock prediction models^21^. In a similar study in 2019, Chung and Shin optimized the kernel size, kernel window, and pooling window size for CNN^22^. In addition, GA has been used to determine appropriate hyperparameters and input data sizes for Generative Adversarial Networks (GANs) in stock prediction by He and Kita in 2021^23^. These studies demonstrate the effectiveness of GA in optimizing the hyperparameters of various deep learning models for stock prediction.

As for the feature selection, many researchers combine GA and other DNN model to reduce input variables and enhance calculation speed by selecting appropriate factors from a large pool of candidate variables. For instance, Chen and Zhou used GA to rank factor importance and select features for a Long Short-Term Memory (LSTM) model, while Milad employed GA as a heuristic approach for selecting relevant features for an Artificial Neural Network (ANN)^24,25^. Li utilized a multilayer GA to select features and reduce high dimensionality in a stock dividend dataset^26^. Recently, Yun revised GA-based selection methods to a two-stage process, using a wrapper method to select important features to avoid the curse of dimensionality, followed by the filter method to select more critical factors^27^.

The challenges associated with the aforementioned methods are distinct:

1. Data decomposition methods are commonly utilized in stock index prediction rather than in the individual stock selection process. The unequal frequencies obtained from time series data pose a significant challenge, hindering the parallel aggregation of decomposed features for individual stocks.
2. Principal Component Analysis (PCA) limitations align with theoretical expectations but often diverge from expected performance in empirical scenarios, demonstrating diminished effectiveness.
3. Existing data augmentation methods are relatively simplistic and exhibit limited efficacy in improving accuracy or alpha effects. These methods mainly expand existing features without notably enhancing their value or informativeness.
4. Tuning a Deep Neural Network (DNN) faces challenges from models with numerous parameters. Even with genetic algorithm integration, computational demands persist. While the genetic algorithm only reduces factors in feature selection.

An encouraging approach is to integrate Genetic Algorithms (GA) principles into the Data Augmentation method. This innovative strategy aims to leverage GA concepts to actively evolve factors and select features from this Genetic Evolved Method. This may lead to more effective feature sets. We'll delve into this proposed novel GA-based data augmentation method in methodology part.

## The proposed deep neural network

In Artificial Intelligence (AI), Deep Neural Network (DNN) falls under the subset of Machine Learning and Neural Network^28^. DNN is based on the artificial neural network (ANN) which contained one or several layers between the input and output layers. In each layer it consists of the same components, and they are neurons, synapses, weights, biases, and functions^29^.

**Figure 1:**
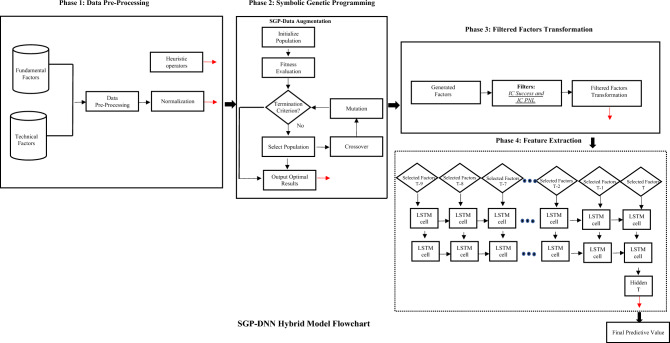
Flowchart of the proposed Deep Neural Network Framework.

The proposed SGP-DNN framework comprises four primary phases: data pre-processing, data augmentation, filtered factor transformation, and feature extraction. As depicted in Fig. 1, during Phase 1, we acquire the dataset from the Alpha Factor Library by S&P Global Market Intelligence, which includes raw fundamental and technical indicators. Instead of conducting feature selection at this stage, our focus is on standard data pre-processing steps such as handling missing values, deleting outliers or noise, and performing feature normalization using the z-score method. Concurrently, we define heuristic formulas to aid the subsequent SGP programming phase.

In Phase 2, SGP operates similarly to a conventional Genetic Algorithm, involving steps like selection, crossover, and mutation. However, it notably differs by utilizing both heuristic generators and normalized features from phase 1 as inputs. A comprehensive explanation of SGP will be provided in Section "data augmentation: symbolic genetic programming". The outcomes of phase 2 consist of optimal features developed through the evolutionary process of SGP, tailored to its specific customized fitness function.

After Phase 2, a set of optimal features (augmented data) is obtained. Phase 3 involves defining rules for feature filtering, considering both their fitness for the model's function and their relevance to the prediction target. These filtered features then undergo transformation to ensure they are of a suitable size and sequence for subsequent use in the LSTM model. The output of Phase 3 comprises the filtered features arranged in an appropriate sequence, in this instance, with lags of 15 days.

The last phase (4) revolves around feature extraction using a DNN model. Specifically, we utilized the Long Short-Term Memory (LSTM) method to discern non-linear patterns, aiming to enhance the accuracy of stock return prediction. Selected features, arranged in sequence from Phase 3, are inputted into a two-layered LSTM model and utilize the final hidden layer output for the final prediction. However, in the feature extraction phase, we also experiment the filtered factors with Multi-Layer Perceptron (MLP). The objective is to observe whether LSTM or MLP could handle the raw data in the extracting pattern according to the nature of feature source (fundamental or technical indicators). A detailed explanation of the DNN model selection process will be presented in Section "Feature extraction method: LSTM vs MLP".

The effectiveness for classification of the prediction from phase I to phase IV is measured using Accuracy, Precision, Recall, Rank Information Coefficient (Rank IC) and Information Ratio of IC (ICIR) as performance (sensitivity) metrics for cross-sectional price change prediction, as demonstrated in Eqs. 1 to 5 In the next Section, we present the discussion on the dataset, software and hardware used in this study, as well as the elaboration on phase I, data augmentation and phase II, feature extraction.

Rank Information Coefficient (Rank IC) serves as a pivotal tool for appraising predictive model efficacy, particularly in portfolio formation during stock selection across a range. This metric evaluates the correlation in rankings between predicted scores of diverse securities and their realized returns, prioritizing relative rankings over precise predictions. It notably facilitates cross-sectional selections aimed at securing alpha or risk-adjusted returns for portfolios. Worth noting is that the sign of Rank IC holds less significance than its magnitude. A positive Rank IC suggests that higher stock values anticipate relatively larger returns, while a negative Rank IC signifies that lower stock values predict larger returns. Additionally, the Information Ratio of Rank IC (ICIR) parallels the Sharpe ratio for a portfolio, providing further insights into its performance.

1 $${\text{Accuracy}}=\frac{Number\, of\, correct\, predictions}{Total\, number\, of\, predictions}$$

2 $${\text{Precision}}=\frac{True\, Positives}{True\, Positives+False\, Positives}$$

3 $${\text{Recall}}\frac{True\, Positives}{True\, Positives+False\, Negatives}$$

4 $$\mathrm{Rank\, Information\, Coefficient}\left(\mathrm{Rank IC}\right)=\frac{\sum_{i=1}^{n}({Rx}_{i}-\overline{Rx})({Ry}_{i}-\overline{Ry})}{\sqrt{\sum_{i=1}^{n}{({Rx}_{i}-\overline{Rx})}^{2}}\sqrt{\sum_{i=1}^{n}{({Ry}_{i}-\overline{Ry})}^{2}}}$$

(R denoted Rank).

5 $$\mathrm{Information\, ratio\, of\, IC}({\text{ICIR}})=\frac{{\text{IC}}}{\mathrm{Standadized\, Deviation\, of\, IC}}$$

### Dataset, software and hardware

In this study, two types of data were utilized during the experiments: fundamental indicators and technical indicators. Fundamental indicators comprise data derived from three types of financial statements, namely the balance sheet, profit and loss report, and cash flow report. On the other hand, technical indicators are based on price and volume, providing users with patterns of momentum and reversal. Prior to processing the data using the proposed method, an analysis based on Rank IC was conducted. Rank IC describes the correlation between predicted and actual stock returns, thereby indicating the degree of alignment between the analyst's fundamental and technical forecasts and the actual financial results. The Information Coefficient (Rank IC) is a numerical measure that ranges from 1.0 to − 1.0. A value of − 1 indicates a perfect negative relationship between the analyst's forecasts and the actual results, while a value of 1 indicates a perfect positive match between the forecasts and the actual results. This metric is highly important when making informed investment decisions, especially in the evaluation of cross-sectional stock returns forecasting. Typically, an information ratio of IC (ICIR) within the range of 0.40 to 0.60, and Rank IC values exceeding 5% in absolute terms, are considered highly favorable in this context.

**Figure 2:**
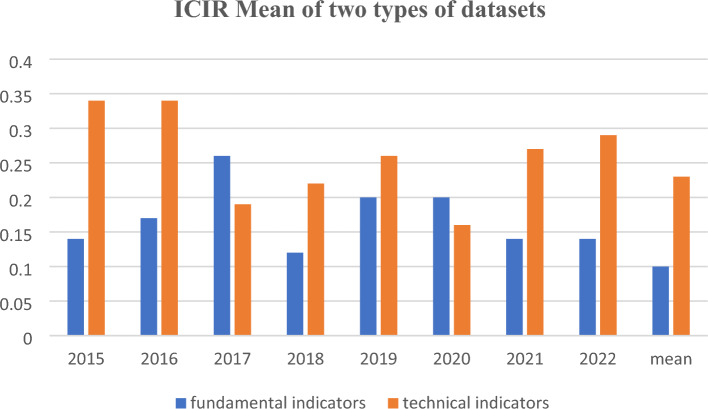
ICIR mean of the dataset.

The data used in this study is dataset of The Alpha Factor Library by S&P Global Market Intelligence^30^, which includes explainable factors for all A-listed stocks (around 4500 listed companies) in the Shanghai and Shenzhen Stock Exchange Market, including fundamental and technical indicators. The appendix contains a comprehensive description of both types of quantitative indicators (304) and their corresponding Rank IC values from 2015 to 2022. Table 1 presents the average Rank IC (Information Coefficient) of two specific type of quantitative indicators, while Fig. 2 illustrates the ICIR (Information Coefficient Information Ratio) of these indicators.

**Table 1:** Rank IC mean of the dataset.

Name of datasets	IC Mean of two types of datasets								
	2015 (%)	2016 (%)	2017 (%)	2018 (%)	2019 (%)	2020 (%)	2021 (%)	2022 (%)	Mean (%)
Fundamental indicators	0.92	1.06	1.63	0.79	1.28	1.36	1.09	1.16	0.65
Technical indicators	4.33	3.75	2.34	2.66	2.81	1.99	3.44	3.73	2.82

For the data preparation and pre-processing, Python 3.8 was employed along with the numpy and pandas packages. The design of DNN models, including LSTM and MLP, was achieved using KERAS 2.4, a package based on Google TensorFlow 2.4. The Symbolic Genetic Programming (SGP) was implemented using the gplearn 0.0.2 package in Python. While the DNN network was trained on NVIDIA GPUs, the remaining models, such as SGP part, were trained on a CPU cluster. Detailed information regarding the software and hardware specifications utilized can be found in Table 2.

**Table 2:** Descriptions on the software and hardware.

Item	Descriptions	Numbers
CPU	Intel(R) Xeon(R) Gold 6248R CPU @ 3.00 GHz	96
RAM	503G	
GPU	GeForce RTX 3090	2
System	Ubuntu 20.04.2 LTS	
Python Version	Python 3.8.5	
Keras Version	2.4.3	
gplearn Version	0.0.2	
Tensorflow Version	2.4.0	

The main aim of this study is to anticipate and forecast changes in cross-sectional stock prices. The target variable is categorized into two statuses: a value of 1 signifies a stock return higher than the medium of cross-sectional stock returns, while a value of 0 indicates a stock return lower than the medium of cross-sectional stock returns over short-term intervals.

The research investigates standard timeframes frequently used in stock predictions, spanning short-term intervals of 5 days (1 week), 10 days (2 weeks), and long-term intervals of 20 days (1 month). This study integrates short-term technical indicators and long-term fundamental indicators. Consequently, the 5-day forecasting period is chosen to assess prediction accuracy. By amalgamating these varied features, the study aims to provide accurate forecasts regarding stock price changes, specifically determining whether they will surpass or fall below the medium of cross-sectional stock prices within the defined 5-day window.

### Data augmentation: symbolic genetic programming

The second step of the proposed DNN framework is to investigate the Genetic Algorithm (GA) in the data augmentation phase. Based on literature, Genetic Algorithms are a type of learning algorithm, that would result in a better neural network by crossing over the weights of two good neural networks. This algorithm could also generate and evaluates consecutive generations of humans in order to achieve optimization objectives. The algorithm creates mutation from the stock related indicators by randomly changing the chromosomes or genes of the individual parents. In this situation, GA can be complicated and costly when implemented on the stock related indicators which is nonlinear and having lots of noise or outliers. Therefore, to solve the problem of nonlinear type of data, the Symbolic Genetic Programming (SGP) is employed in this study. SGP has several advantages as it evolve by building blocks. In SGP, it employed the regression analysis which is more robust to search the space in finding the best model to fit the given stock return data. Different from GA, SGP find an intrinsic relationship between two or more variables which is hidden. Typically, there are two types of genes that contribute to the generations.

**Figure 3:**
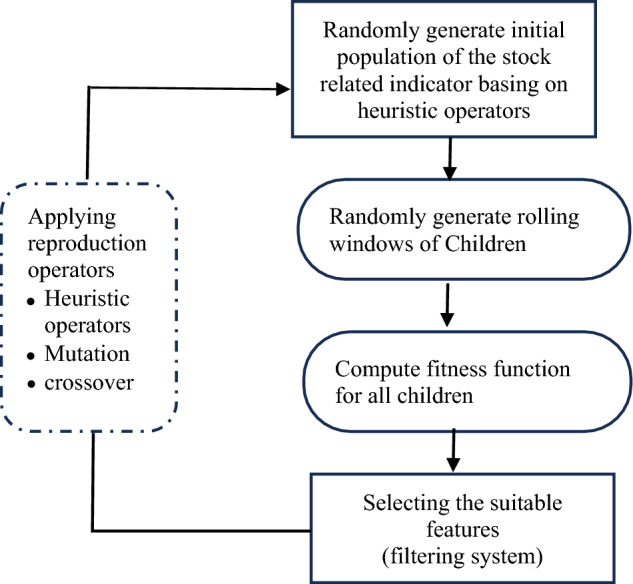
The structure of the proposed Symbolic Genetic Programming.

The first type in the study refers to the input features, while the second type represents the processing operators, encompassing mathematical functions like addition, subtraction, division, and multiplication. Predicting stock price data can be a daunting task, given its complex, dynamic, and non-linear nature. To tackle this challenge, mainstream hedge funds like World Quant, Cubist, and Menelia employ various heuristic operators such as correlation, covariance, and variance. These operators help them analyze and interpret the data, enabling them to make informed investment decisions^31^, as depicted in Table 3, to enhance the analysis and prediction of stock price data. In this study, an improved Symbolic Genetic Programming (SGP) is proposed, which utilizes symbolic tree expressions to handle and solve complex optimization problems, providing greater flexibility. The four-step approach outlined in Fig. 3 is applied to enhance the performance of the SGP.

**Table 3:** Heuristic operators.

The Heuristic operators				
'decay_linear'	'rank_add',	'rank_sub',	rank_mul'	'rank_div'
'ts_max'	'ts_min'	'ts_nanmean'	'ts_prod'	'ts_rank'
'ts_stddev'	'ts_sum'	'ts_corr'	'ts_cov'	'delta'
sign'	'ts_skewness'	'ts_kurtosis'	'ts_max_diff'	'ts_min_diff'
'ts_zscore'	'ts_scale'	'ts_min_max_cps'	'ts_ir'	'ts_median'
'winsorize'	'zscore'	'ts_argmax'	'ts_argmin'	'rank'
'delay'	'sigmoid'	'ts_return'

The first step in our proposed SGP, is to initiate the population of the genes. Here, we introduce the heuristic operators like the Table 3 shows in the reproduction of the genes. To guide the evolution of the genes, we set certain parameters. For instance, we established a probability of 40% for crossover, which involves exchanging genes between two individuals in the population. Additionally, we set a 40% probability for replacement, which involves copying an individual gene in the population. Finally, we assigned a very low probability for three types of mutation to prevent an excessive influx of new input features, which could lead to unpredictability. This helps maintain stability in the incorporation of new genetic material into the population.

Second, we designed and added rolling windows for all heuristic operators to the original SGP. To this end, we randomly generate rolling window seeds between 3 and 20 for rolling window to produce additional symbolic formulas. The third step is to design the fitness function. In this study, calculations are performed to determine the fitness target. In addition to using the original Pearson correlation (Rank IC) between the value of the symbolic formula and future price change as the fitness target, a combined formula will be used. This combined formula takes into consideration both the relatively high cumulated return of the bottom group among all cross-sectional stocks and the maintenance of monotonicity in the cumulated return of k groups based on the order of values in the symbolic formula. By incorporating these factors, the fitness target aims to optimize the performance of the symbolic formula in predicting stock price changes.

The formula is shown from Eqs. (6) to (8) below:

6 $$To{p}_{R}={\text{max}}(TopR-mean\left(totalR\right),FlopR-mean\left(totalR\right))$$

5 $${\text{Monotonicity}}={\text{max}}(\frac{1}{N}\sum_{k=1}^{N}{\text{max}}(0,{Sign(R}_{k}- {R}_{k+1})),\frac{1}{N}\sum_{k=1}^{N}{\text{max}}(0,{Sign(R}_{k+1}- {R}_{k})))$$

7 $$\mathrm{Rank\, Information\, Coefficient}=\frac{\sum_{i=1}^{n}({Rx}_{i}-\overline{Rx})({Ry}_{i}-\overline{Ry})}{\sqrt{\sum_{i=1}^{n}{({Rx}_{i}-\overline{Rx})}^{2}}\sqrt{\sum_{i=1}^{n}{({Ry}_{i}-\overline{Ry})}^{2}}}$$

8 $${\text{Fitness}}={{\text{Top}}}_{{\text{R}}} +{\uplambda }_{1}\times \mathrm{Monotonicity }+{\uplambda }_{2}\times \mathrm{ Information\, }{{\text{Coefficient}}}^{\mathrm{^{\prime}}}$$

$$({\mathrm{Default \lambda }}_{1}=0.4 {\mathrm{Default \lambda }}_{2}=2)$$

After obtaining many symbolic formulas based on the above algorithms, the final amendment for SGP is the filter system for the outcomes. The success ratio of Pearson correlation (Rank IC) and the profit and loss ratio (P&L ratio) of Pearson correlation from Eqs. (9) to (10) will be employed to select the final synthetic symbolic formulas generated by the SGP model. These above two ratios will also be used as metrics for the experiment part

9 $$\mathrm{Success\, Ratio\, of\, Rank\, IC }\left(\mathrm{IC\, success\, Ratio}\right)=\frac{Numbers\, of\, Correct\, Pearson\, IC}{Total\, num\, of\, Pearson\, IC}$$

10 $$\mathrm{Profit\, and\, Loss\, Ratio\, }\left(\mathrm{IC\, PNL}\right)=\frac{Mean(\left|Pearson\, IC\right|)}{Standard\, devation(Pearson\, IC)}$$

### Feature extraction method: LSTM vs MLP

The final step in the proposed hybrid DNN framework involves extracting features from the augmented selected data obtained through the SGP process. Feature selection is carried out by creating a Hybrid DNN model that accommodates individual data sources based on their specific characteristics.

**Figure 4:**
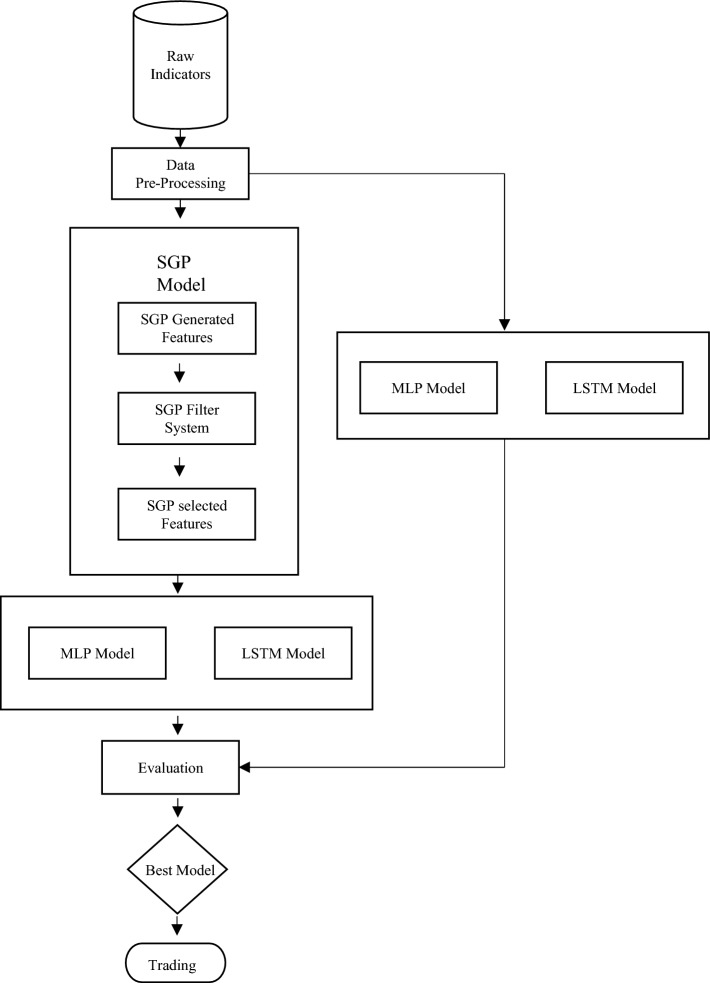
Feature extract method: LSTM vs MLP.

Since the development of DNN, the Multiple Layer Perceptron (MLP) was initially introduced as a basic supervised learning algorithm with multiple layers, each consisting of several neurons. However, MLPs have a significant drawback in their ability to handle sequence or time series data effectively. This limitation poses a crucial challenge in stock return forecasting, which heavily relies on the historical states of stocks, following a Markov Chain. To address this issue, a more suitable approach is to utilize the LSTM (Long Short-Term Memory) model, which falls under the category of Recurrent Neural Networks (RNN). LSTMs are specifically designed for sequence modelling tasks and overcome the limitations of MLP. Both LSTM and MLP models are chosen for comparisons, as shown in Fig. 4.

In the first step, the original indicators are either inputted into the SGP model (as depicted in Fig. 4) to obtain selected features, which are then fed to the MLP or LSTM model. Alternatively, the original indicators can be directly fed into the MLP or LSTM model for comparison.

The performance of the four experiments is evaluated using metrics such as Rank IC and ICIR to determine the best model based on the dataset's unique characteristics. The optimization goal for all network settings is to minimize Mean Squared Error (MSE), while the performance quality is assessed using Rank IC and ICIR as metric indicators. Finally, the trained network is used to recognize feature patterns, and based on the Enhanced SGP-DNN Framework, simple trading rules suitable for the stock market are formulated. These rules are then ‘backtested’ in stock trading scenarios.

**Figure 5:**
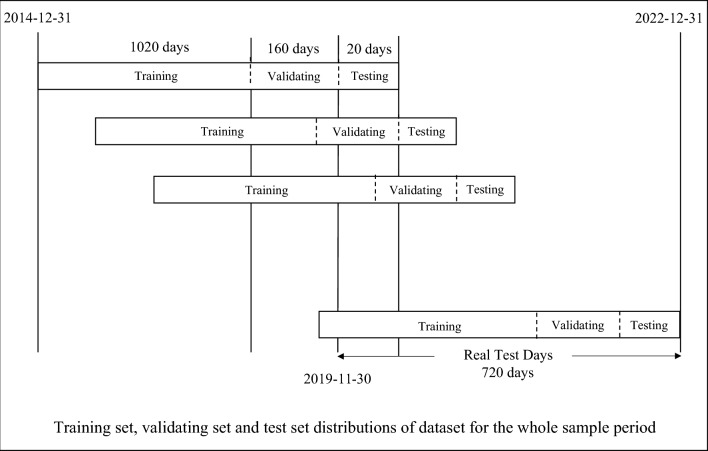
Train/validation/test set for rolling window.

To ensure simulating the real stock investing and considering the ‘backtest’^32^, the forward rolling window and the segmentation prediction method were followed, the specific details are illustrated in Fig. 5. The whole sample period will be divided into three parts, in the training part, the dataset length is 1020 days which is used to update the model parameters. As for validation part, we use 160 days for tunning and the test part is 20 days and as a result the rolling window is also set as 20 days. The ratio of training set, validation set is taken as 8.5:1 and the real test days is 720 days from 2019-11-30 to 2022-12-31-resulting in a total of 36 non-overlapping trading periods.

### Forecasting, ranking, and trading

The SGP-DNN model utilizes available information prior to time t to forecast the future price change of each stock. Its objective is for each stock to surpass the average price changes observed in the cross-sectional market during the subsequent period t + 1. To achieve this, the model ranks all cross-sectional stocks (4500 in total) in ascending order based on the predicted return by SGP-DNN for the next period. The highest-ranked stocks form the top group, and historically, we have divided the entire cross-sectional stocks into 10 groups, each containing 450 stocks. This ranking score serves as a basis for long only portfolio construction.

Long-Only Portfolio Strategy: The Long-Only Portfolio Strategy focuses on taking long positions in the top k stock portfolios, which are then held for a single period (t + 1). To gauge the effectiveness of this strategy, we will compare its performance against the CSI 300 and CSI 500 benchmarks (denoted as Excess R above 300 and Excess R above 500). These benchmarks represent broad-based indexes in the Chinese stock market. Moreover, we will also consider the average performance of an equal-weighted portfolio as a third performance benchmark (denoted as Excess R above average), the sharp ratio of Excess R above average (Sharp Ratio) will be also measured as the metrics in experiment part.

## Experiments with five classical DNN frameworks for comparison

In this study, two types of raw data—fundamental and technical indicators—are utilized to evaluate the Neural Network's performance. The objective is to experiment with both datasets and identify potential discrepancies in outcomes.

Preceding this analysis, an extensive comparative test was conducted. The subsequent metrics will illustrate comparisons among several models: classical Thomas Fischer's LSTM model, Ghosh’s three features LSTM model, single LSTM model with raw features, PCA-LSTM model with raw features, and proposed SGP-LSTM model with raw features.

**Figure 6:**
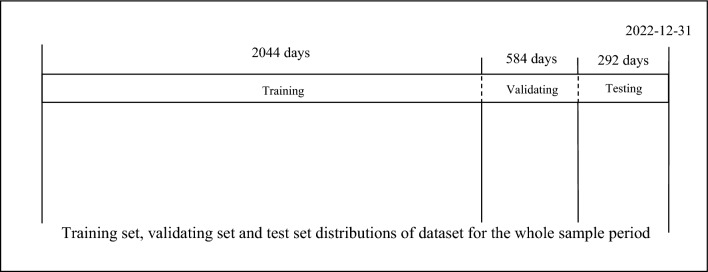
Train/validation/test set for the whole sample period.

Figure 6 displays our comparison among these 5 models, utilizing a train/validation/test split without employing rolling window or segmentation prediction methods for direct comparisons. The entire sample period was divided into a 70% train set, a 20% validation set, and a 10% test set, enabling a thorough evaluation of the comparison metrics.

**Figure 7:**
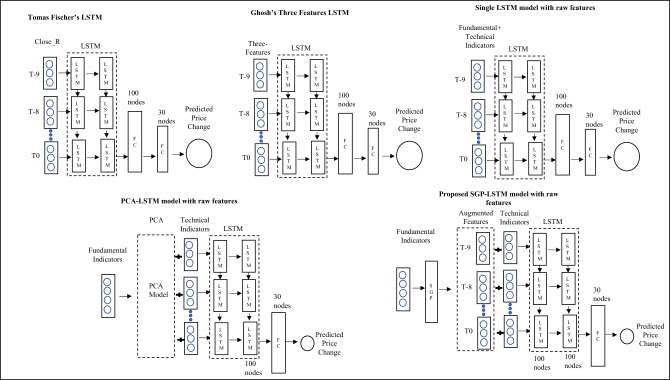
The DNN frameworks comparisons for 5 models.

In Fig. 7, the study employs Thomas Fischer’s LSTM and Ghosh’s Three Factors LSTM models for cross-sectional stock selection using Chinese stock data. Additionally, a PCA-LSTM model was developed based on Zhang et al.'s methodology^17^. The figure also presents the Single-LSTM model and the proposed SGP-LSTM model, both utilizing raw features.

The examination of metrics across five models in Table 4 unveils intriguing discoveries. Thomas Fischer's LSTM model notably improves accuracy, achieving 53.8% in the Chinese stock market, surpassing its previously reported 51.4% accuracy in the US stock market (1992 to 2015). Unexpectedly, despite incorporating two additional features into Ghosh's model, there's no observable accuracy enhancement within the Chinese stock market (53.2% for Ghosh compared to Thomas' 53.8%).

**Table 4:** Metrics of Comparisons among different models.

Case	Rank_IC (%)	Accuracy (%)	Precision for positive (%)	Recall for positive (%)	AUC (%)	forecasted positive num/forecasted positive num
PCA-LSTM model with raw features	9.0	53.5	52.2	62.3	53.5	1.41 (3180:2250)
Proposed SGP-LSTM model with raw features	9.3	53.7	53.2	47.8	55.4	0.79 (2073:2629)
Single LSTM model with raw features	7.3	52.8	51.7	58.4	54.2	1.24 (2635:2120)
Ghosh’s three features LSTM	10.7	53.2	51.5	82.7	56.2	**3.75 (3727:995)**
Thomas Fischer’s LSTM	10.6	53.8	52.1	71.5	56.1	**2.05 (3177:1547)**

In the related work section, I highlighted the persistent trend of negative accumulated alpha observed in both Thomas and Ghosh's LSTM models over the past decade. Table 5's data reveals a compelling pattern: the ratio between forecasted positive and negative outcomes exceeds 2, notably peaking at 3.75 in Ghosh's model. This underscores a consistent bias toward predicting positive outcomes. For instance, in Ghosh's model, out of over 4700 stocks, 3727 are forecasted as positive compared to 995 forecasted as negative, showcasing a recurring inclination to predict individual stock returns surpassing the median of all sectional stocks. This trend may significantly contribute to the negative accumulated alpha observed within the top group portfolio (trading rules for the 10 groups outlined in Section "Forecasting, ranking, and trading"), to be further explored in Section "Proposed models comparison with Fisher’s LSTM model, Ghosh’s three features LSTM model" through experimentation. A similar pattern is evident in Thomas's model. Conversely, the other three models exhibit a more balanced distribution between positive and negative forecasted outcomes.

**Table 5:** The metrics of fundamental indicators for DNN with MLP or LSTM.

Fundamental indicators				
Metric	SGP-MLP	SGP-LSTM	MLP	LSTM
2020				
Rank IC	− 7.15%	− 6.70%	− 2.95%	− 2.46%
ICIR	− 4.20	− 3.90	− 2.79	− 2.25
Excess R above 300	− 7.87%	− 5.83%	1.39%	− 3.53%
Excess R above 500	− 3.35%	− 1.20%	6.28%	1.15%
Excess R above average	0.27%	2.39%	10.01%	4.84%
Sharp ratio	0.04	0.34	1.84	1.01
2021				
Rank IC	− 7.13%	− 7.25%	− 1.04%	− 2.13%
ICIR	− 4.24	− 5.58	− 0.80	− 2.72
Excess R above 300	45.71%	41.29%	31.96%	23.85%
Excess R above 500	21.40%	17.49%	9.92%	2.91%
Excess R above average	12.77%	8.83%	1.62%	− 4.61%
Sharp ratio	1.67	1.38	0.27	− 0.91
2022				
Rank IC	− 9.86%	− 9.99%	− 1.58%	− 4.07%
ICIR	− 6.11	− 7.32	− 1.26	− 5.08
Excess R above 300	35.99%	34.59%	20.57%	20.60%
Excess R above 500	34.54%	33.12%	18.96%	19.16%
Excess R above average	19.05%	17.84%	5.27%	5.46%
Sharp ratio	2.77	3.09	0.69	1.26

Table 4 showcases the SGP-LSTM model achieving the highest accuracy rate of 53.70% in the test set. However, upon comparing accuracy and Rank IC, the Single-LSTM model (52.80%, 7.3%) appears less effective compared to the PCA-LSTM model (53.50%, 9.0%), suggesting an advantage of PCA-LSTM in predictive effectiveness over Single-LSTM. Notably, substituting PCA with SGP led to an improvement in accuracy and Rank IC from (53.5%, 9.0%) to (53.7%, 9.3%). These results signify that SGP-LSTM outperforms PCA-LSTM, validating the efficacy of data augmentation or decomposition methods.

Additionally, considering both the balanced distributions for positive and negative forecasted outcomes and the Rank-IC, the Proposed SGP-LSTM model demonstrates superiority over the other four models. Further exploration of this superiority will be detailed in Section "Proposed models comparison with Fisher’s LSTM model, Ghosh’s three features LSTM model" through experimentation.

After completing experiments on the five models using machine learning metrics, our next step involves a more in-depth exploration of the alpha effect employing the Proposed SGP-LSTM model. This exploration will utilize two distinct sets of raw features processed through rolling windows, following the trading rules outlined in Section "Forecasting, ranking, and trading".

In our stock trading experiment, we conducted 'backtests' spanning 720 days. The prediction horizon was fixed at 5 days, and we implemented a rolling cycle of 20 days. This setup allowed the hybrid model to optimize its parameters every 20 days, utilizing the 1180 data points mentioned earlier. These optimized parameters remained consistent for the subsequent 20-day period. Additionally, every 5 days, the model ranked its stock prediction values, selecting the top 10% (450 stocks) for portfolio construction. Equal weights were assigned for buying and holding, and stocks with limitations were excluded to address potential trading issues. This approach ensured systematic assessment while constructing the portfolio based on the model's predictions within this timeframe.

Following the procedure of Proposed SGP-DNN model in Section "The proposed deep neural network", we also nee to examine the efficacy of SGP when integrated with MLP and LSTM, thereby assessing the suitability of SGP in conjunction with both methods. The experiment will be segmented into two main sections: Section "Experiment with fundamental indicator" will elaborate on experiments using fundamental indicators, while Section "Experiment using technical indicator." will focus on experiments employing technical indicators. This structured approach aims to comprehensively explore the impact and potential of integrating SGP within different feature processing methods.

### Experiment with fundamental indicator

We execute the experiment with the fundamental indicator. This experiment is to observe 8 metrics of the cross-sectional stock return prediction based on the fundamental indicator, whether the integration of SGP give improvement or vice versa. First, we experiment the fundamental indicator directly using the MLP method. Then followed by experimenting it using LSTM method. This experiment is without integration of SGP. To observe the capability of SGP, we executed an experiment based on the using the LSTM and MLP respectively with the integration of SGP method. Table 5, illustrates the results of the experiments conducted, where the LSTM or MLP is integrated with SGP, is labelled as SGP-MLP and SGP-LSTM respectively. While the results obtained without the integration of SGP is shown in the column labelled as MLP and LSTM respectively.

**Figure 8:**
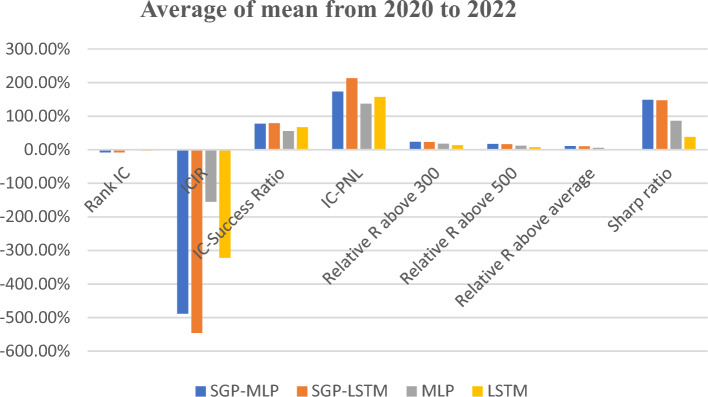
The metrics from 2020 to 2022 for fundamental indicators.

Table 5 shows the results executed from the experiment for data in the year of 2020 to 2022. Whereas Fig. 6 summarize the data from 2020 to 2022 based on its average mean. Based on the results shown in Fig. 8, the results indicate that when the raw fundamental indicators were used as input for LSTM or MLP models, the average IC values were − 1.85% and − 2.88%, respectively. The average value of IC in this situation is considered low whereby the ideal average value should be above 8%. While the average value for ICIR were − 1.55 and − 3.22, respectively. This value for cross-sectional stock price change prediction is considered average or acceptable. The ideal value for ICIR is above 3. However, after integrating the models with the SGP algorithm, the IC absolute values increased to 8.05% for MLP and 7.98% for LSTM which is considered as ideal outcome.

The SGP-LSTM model attained the highest average value of − 5.46 for ICIR, surpassing the performance of other models. It exhibited superior results in terms of IC-success ratio and IC-PNL, with values of 78.91% and 2.13, respectively. Furthermore, both the SGP-LSTM and SGP-MLP models showcased notable advantages over the single DNN models by employing a straightforward rule-based strategy for a long-only approach. Specifically, the SGP-LSTM model demonstrated a Excess R exceeding the CSI 300 index by 22.35% and surpassing the CSI 500 index by 16.26%. Moreover, it achieved an Excess R above the average by 9.89% per year, positioning it among the top 10% of mutual fund managers in China.

### Experiment using technical indicator.

**Figure 9:**
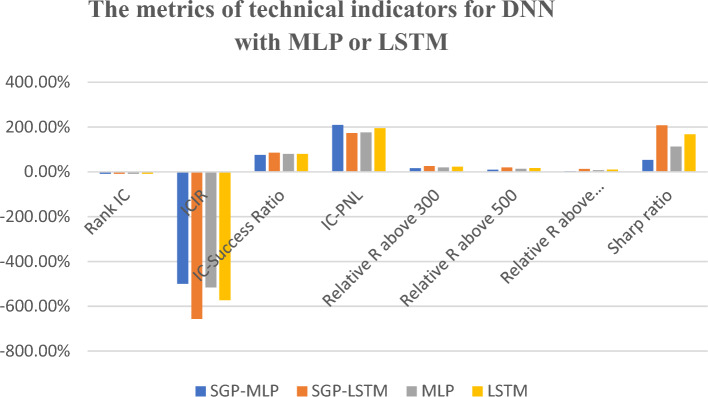
The metrics of technical indicators for DNN with MLP or LSTM.

In contrast, according to the findings presented in Table 6 and Fig. 9, SGP-MLP or SGP-LSTM does not demonstrate significant advantages over single DNN models when it comes to technical indicators. On average, the single LSTM model for technical indicators produced the best results in terms of normal metrics such as IC, ICIR, and IC-success, with percentages of -8.64%, -6.561, and 85.71% respectively (the original IC mean of technical indicators is 2.82% and ICIR mean is 0.23 from Table 1 and Fig. 2). When comparing the performance of the two single DNN models in relation to a simple rule-based strategy, the LSTM model outperformed the MLP model. This could be attributed to the fact that technical indicators represent sequential time series data, which is better suited for the LSTM model, as explained in the methodology section. Notably, when considering a long-only strategy, the LSTM model exhibited a significantly higher Excess R above average at 13.28%, compared to the MLP model's 3.94%.

**Table 6:** The metrics of technical indicators for DNN with MLP or LSTM.

Technical indicators				
Metric	SGP-MLP	SGP-LSTM	MLP	LSTM
2020				
Rank IC	− 8.07%	− 7.68%	− 8.08%	− 9.49%
ICIR	− 4.575	− 5.313	− 4.345	− 6.970
Excess R above 300	− 5.06%	− 4.28%	− 7.65%	8.98%
Excess R above 500	− 0.41%	0.59%	− 3.22%	14.25%
Excess R above average	3.32%	4.05%	0.48%	18.60%
Sharp ratio	0.520	0.701	0.072	3.098
2021				
Rank IC	− 7.76%	− 8.14%	− 7.84%	− 7.74%
ICIR	− 4.741	− 5.684	− 4.978	− 5.876
Excess R above 300	41.55%	45.18%	35.25%	42.83%
Excess R above 500	17.58%	20.61%	12.29%	18.61%
Excess R above average	9.25%	11.76%	4.41%	9.92%
Sharp ratio	1.315	2.062	0.586	1.591
2022				
Rank IC	− 9.26%	− 8.92%	− 9.49%	− 8.68%
ICIR	− 6.033	− 5.899	− 5.541	− 6.540
Excess R above 300	26.00%	31.07%	22.11%	25.87%
Excess R above 500	24.55%	29.54%	20.44%	24.45%
Excess R above average	10.15%	14.39%	6.56%	9.97%
Sharp ratio	1.551	2.268	0.920	1.612

**Figure 10:**
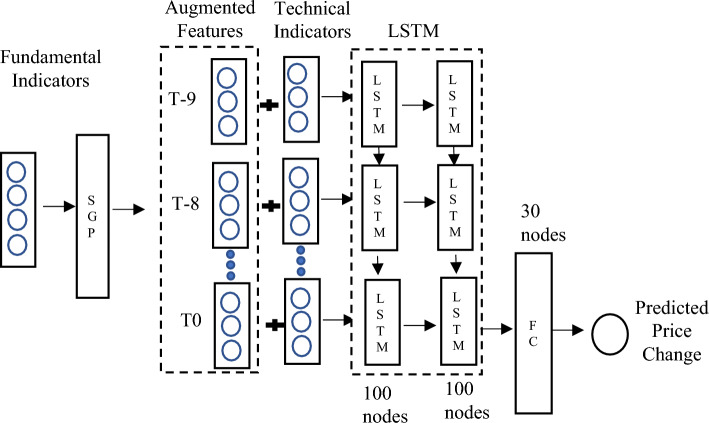
The proposed SGP-DNN framework for raw indicators.

Based on the experiments conducted earlier, we could summarize that the fundamental indicator will achieve the best result, when the indicators are fed into SGP algorithm, while the technical indicator will achieve the best result without integrating the SGP but directly through LSTM technique. Therefore, we design a new DNN framework that could work well with both fundamental and technical indicators. Figure 10 below illustrates the proposed DNN framework where both fundamental and technical indicators are fed as the raw data. The explanation on the experiment on this proposed framework will be discussed in the next section.

### Experiment using fundamental and technical features.

Based on Fig. 10, the fundamental indicators are first fed as the raw data into the framework. As mentioned earlier, the results shows better when SGP is integrated with LSTM or MLP. Therefore, the fundamental indicators are processed based on SGP and the output is being an input for the Phase I, the augmentation phase. The output for the augmentation phase is combined with the technical indicators to be an input for the Phase II, the feature selection. Here, only LSTM is utilized as from the experiment executed earlier, LSTM outperformed the MLP in terms of a better results. Two layers of LSTM are performed with 100 nodes each, where the final feature selection is only 30 notes for the price changes prediction. In Section "Experiment using fundamental and technical features.", we present the results based on the experiments conducted using the new proposed framework as shown in Fig. 10.

**Figure 11:**
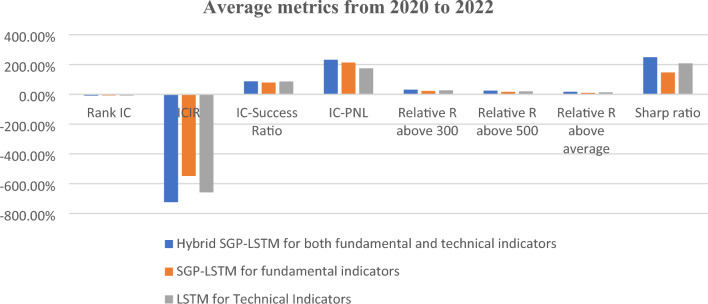
The metrics based on the proposed SGP-LSTM framework for the average mean data.

According to Table 7 our hybrid model showcased a significant improvement of 1128% in information coefficient (IC) and an impressive surge of 5360% in IC information ratio (ICIR) when applied to fundamental indicators. For technical indicators, the hybrid model achieved a commendable 206% increase in IC and a remarkable surge of 2752% in ICIR. According to Table 8 and Fig. 11, the proposed SGP-LSTM model attained a rank IC value of 9.24% and an ICIR of 7.24 for a five-day prediction horizon.

**Table 7:** The metrics based on the proposed SGP-LSTM framework.

	Original rank IC	Hybrid model IC	Original ICIR	Hybrid model ICIR
Fundamental indicators	0.65%	7.98%	0.1	5.46
Technical indicators	2.82%	8.64%	0.23	6.56
proposed SGP-DNN		9.24%		7.24

**Table 8:** The metrics based on the proposed SGP-DNN framework.

Year	Metric	Hybrid model for quantitative indicators	Hybrid model for quantitative indicators individually	
	Metric	Hybrid SGP-LSTM for both fundamental and technical indicators	SGP-LSTM for fundamental indicators	LSTM for Technical Indicators
2020	Rank IC	− 10.78%	− 6.70%	− 9.49%
	ICIR	− 8.71	− 3.9	− 6.97
	Excess R above 300	13.53%	− 5.83%	8.98%
	Excess R above 500	19.13%	− 1.20%	14.25%
	Excess R above average	21%	2.39%	18.60%
	Sharp ratio	3.26	0.34	3.1
2021	Rank IC	− 8.64%	− 7.25%	− 7.74%
	ICIR	− 7.03	− 5.58	− 5.88
	Excess R above 300	52.46%	41.29%	42.83%
	Excess R above 500	26.71%	17.49%	18.61%
	Excess R above average	12%	8.83%	9.92%
	Sharp ratio	1.00	1.38	1.59
2022	Rank IC	− 10.34%	− 9.99%	− 8.68%
	ICIR	− 8.99	− 7.32	− 6.54
	Excess R above 300	26.04%	34.59%	25.87%
	Excess R above 500	24.72%	33.12%	24.45%
	Excess R above average	18%	17.84%	9.97%
	Sharp ratio	2.95	3.09	1.61

### Proposed models comparison with Fisher’s LSTM model, Ghosh’s three features LSTM model

This section focuses on comparing the alpha effect of the top group portfolio among the Proposed SGP-LSTM model, Thomas Fischer's, and Ghosh's models. The rolling windows test, executed with identical settings between Section "Feature extraction method: LSTM vs MLP" and Section "Forecasting, ranking, and trading", aimed to compare the performance of the proposed SGP-LSTM model against Thomas Fischer's and Ghosh's LSTM models. This comprehensive analysis involved evaluating metrics for both the DNN model and the portfolio's risk-adjusted accumulated return.

Table 9 presents the outcomes derived from the 2020 to 2022 test set, delineating the average metrics for the three models scrutinized in this study. As expounded in Section "Experiments with five classical DNN frameworks for comparison", limitations regarding the uneven distribution of forecasted positive and negative outcomes are evident for both the Thomas Fischer and Ghosh models. Specifically, as highlighted in Table 9, the precision metric exhibits poor performance for Thomas and Ghosh models, registering values of 52.1% and 51.5%, respectively, compared to 53.07% for the Proposed SGP-LSTM model. Despite their higher Rank IC values, as anticipated in Section "Experiments with five classical DNN frameworks for comparison", all models resulted in negative Excess Return (alpha). Ultimately, the Proposed SGP-LSTM model demonstrated a 16.38% Excess Return (alpha) for the test set, accompanied by an information ratio of around 2.66.

**Table 9:** The mean of metrics comparisons among three models.

Metric	Thomas Fischer’s LSTM	Ghosh’s LSTM	Proposed SGP-LSTM Model
Rank IC	10.6%	10.7%	9.24%
Accuracy	53.8%	53.2%	54.17%
Precision	52.1%	51.5%	53.07%
Recall	71.5%	82.7%	57.13%
Excess return	− 6.34%	− 6%	**16.38%**
Excess volatility	2.93%	2.83%	6.15%
Information ratio	− 2.19	− 2.10	2.66

**Figure 12:**
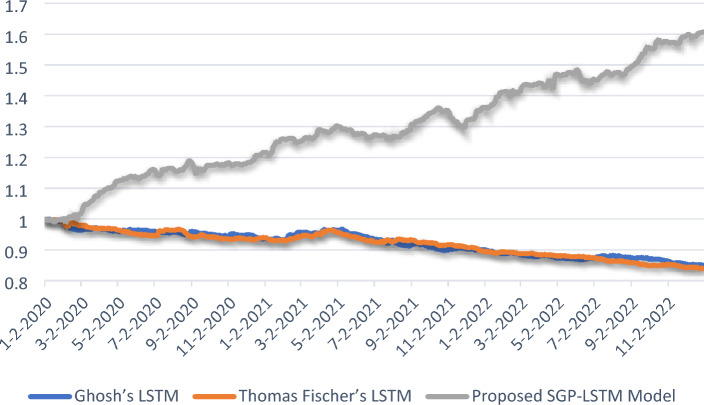
Comparisons of excess R above average.

Furthermore, Fig. 12 showcases the cumulative performance comparison of Excess Returns over the average return of all stocks within three long-only portfolios: Ghosh’s LSTM model, Thomas Fischer’s LSTM model, and our proposed enhanced SGP-LSTM model, which yields the most favorable outcomes. Over the three-year out-of-sample period, it achieves a relative annual return of 16.38% and accumulates a total return of 57.62%. In contrast, despite exhibiting relatively high accuracy rates, the accumulated excess returns of Thomas Fischer and Ghosh remain negative.

**Figure 13:**
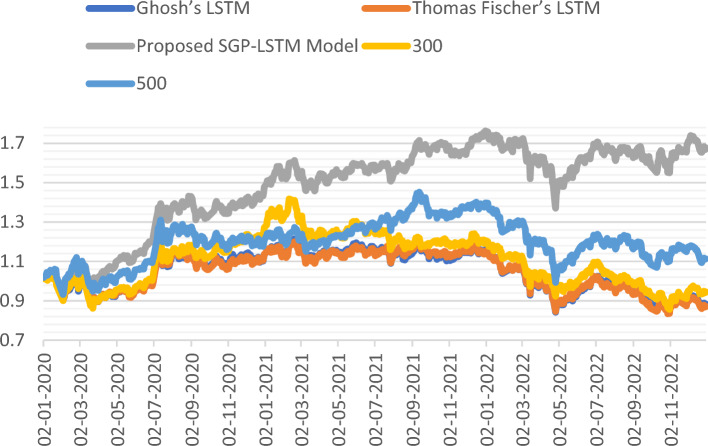
Comparison of the cumulative return curves.

Figure 13 presents a comparison of the cumulative return curves for the proposed SGP-LSTM model portfolio and two broad-based indices, as well as Ghosh’s LSTM and Thomas Fischer’s LSTM portfolio, during the period of 2020–2022. The results clearly demonstrate that the proposed model outperformed the average portfolio, as well as the CSI 300 and CSI 500 indices. Notably, the SGD-LSTM hybrid model exhibited significant outperformance compared to the CSI 300 index, the CSI 500 index as well as two benchmark portfolios, as shown in Fig. 13. Over the span of three years, the proposed SGP-LSTM model accrued an accumulated R of about 67.75%. In contrast, the CSI 500 achieved 11.32% in accumulated R, whereas both CSI 300 and the two benchmark portfolios accumulated negative returns.

## Conclusion

This paper introduced a methodology to enhance the cross-sectional stock return prediction by utilizing Symbolic Genetic Programming (SGP) for input generation and integrating it with Deep Neural Network (DNN) models. The study demonstrated significant improvements in prediction, outperforming popular market indexes. A hybrid model combining SGP with Long Short-Term Memory (LSTM) showcased superior performance, consistently surpassing market returns a simple rule-based strategy based on the proposed hybrid SGP-LSTM model outperforms major Chinese stock indexes, generating average annualized excess returns of 31.00%, 24.48%, and 16.38% compared to the CSI 300 index, CSI 500 index, and the average portfolio, respectively. The findings highlight the potential of the proposed approach in generating profitable investment strategies and provide insights into addressing challenges in data integration and feature engineering.

This study focused solely on financial time series data, which is known for its high autocorrelation. However, recent research has explored the incorporation of diverse data sources such as social media data, news, macroeconomic data, and high-frequency data. Moreover, the proposed hybrid SGP-DNN model could benefit from additional optimization targets, such as Excess Return of top groups or monotonicity of ten groups of target stocks, instead of solely relying on MSE as the optimization goal. Additionally, recent advancements in reinforcement learning or generative adversarial networks (GANs), such as ChartGPT application, have been suggested to be combined with hybrid DNN models. Therefore, it could be worthwhile to consider supplementing the suggested hybrid SGP-DNN model with GANs or reinforcement learning techniques to leverage multi-source information and improve prediction performance.

## Data Availability

The data used in this study is dataset of The Alpha Factor Library by S&P Global Market Intelligence (https://www.marketplace.spglobal.com/en/datasets/alpha-factor-library-(3), which includes explainable factors for all A-listed stocks (around 4500 listed companies) in the Shanghai and Shenzhen Stock Exchange Market, including fundamental and technical indicators. The data is exclusive and purchased by Pingan Group at the price of 50,000 US dollars per year for research and the following Diagram described the database descriptions from the link above. And if required, I could provide the sample of dataset for instance the fundamental and technical factors(400 factors) for 4500 listed stocks from China Stock market for one month.

## Appendix

1. The fundamental indicators and its original Rank IC from 2015 to 2022.

**Table:**

	2015 (%)	2016 (%)	2017 (%)	2018 (%)	2019 (%)	2020 (%)	2021 (%)	2022 (%)
Val-style	0.50	3.85	5.98	3.53	3.18	2.49	3.31	3.44
LogMktCap	9.94	7.89	− 3.68	1.77	0.47	− 1.17	4.87	3.41
LogMktCapCubed	9.27	7.87	− 3.69	1.77	0.48	− 1.18	4.82	3.37
IndRel_TobinQ	1.04	3.68	3.56	2.12	1.06	− 0.14	3.85	4.27
ROEStddev20Q	− 0.32	0.63	1.16	1.63	2.10	2.86	1.40	2.65
ROAStddev20Q	− 1.40	0.50	1.43	1.27	1.39	2.54	0.91	2.39
CE-style	0.30	0.55	1.64	1.66	2.35	2.56	− 0.50	− 0.45
ShareChg	0.38	1.51	0.72	0.42	0.35	0.51	1.74	1.87
EQ-style	− 1.07	0.42	2.17	1.23	1.91	2.38	− 0.29	0.41
AccrualRatioBS	0.16	2.49	0.39	1.09	− 0.39	− 1.03	1.75	2.17
SusGrwRate	1.52	1.62	− 1.43	0.85	− 0.45	− 0.27	1.82	2.44
AstGrwth	0.75	2.91	− 0.18	1.21	− 1.33	− 2.05	1.99	2.56
HG-style	− 0.11	− 0.29	2.90	0.36	1.95	2.44	− 1.05	− 0.55
IndRel_SusGrwRate	1.76	1.36	− 1.22	0.71	− 0.30	− 0.28	1.45	1.99
3YAvgAnnSalesGrw	0.80	2.34	− 1.47	1.28	− 0.15	− 1.26	1.68	2.24
Sz-style	6.93	2.98	− 5.20	0.36	− 0.53	− 2.35	2.29	0.97
AdjAccruals	− 0.16	1.69	0.78	1.06	− 0.01	− 0.17	0.91	1.33
Chg1YCACL	0.77	0.43	0.80	0.29	0.43	0.32	1.17	0.99
6MAvgChg1MRecC	0.85	0.28	0.93	0.24	− 0.04	0.90	0.36	0.68
CashCycle	− 1.21	0.24	1.63	0.32	1.03	1.27	− 0.10	0.81
ChgDeprCapex	− 1.44	0.22	1.43	0.98	0.91	0.86	0.13	0.66
DA	2.06	0.04	− 1.01	1.24	1.82	0.69	− 0.25	− 0.86
IndRel_AccrualRatioCF	0.15	1.47	− 0.58	0.95	− 0.56	− 0.41	1.21	1.26
UnexpectedRecChg	0.56	0.23	0.41	0.28	0.97	1.20	− 0.37	0.20
AE-style	0.24	− 0.03	1.74	0.80	0.95	1.17	− 0.44	− 1.03
IndRel_CashCycle	− 0.49	1.07	0.69	0.02	0.40	0.92	0.41	0.26
IndRel_DA	1.52	0.25	− 0.44	0.86	1.16	0.90	− 0.45	− 0.57
WCAccruals	− 0.20	1.25	− 0.10	0.59	− 0.23	− 0.37	0.86	1.12
IndRel_WCAccruals	− 0.09	1.14	− 0.11	0.29	− 0.27	− 0.27	0.96	1.18
TotalAccruals	− 1.68	0.52	1.62	0.54	0.41	0.89	− 0.44	0.87
IndRel_AdjAccruals	0.21	0.54	− 0.16	0.65	0.54	− 0.36	0.13	0.83
5YRel_QuickRatio	0.68	0.00	0.69	0.40	− 0.05	− 0.51	0.40	0.40
IndRel_ShareChg	− 0.77	− 0.60	0.80	1.37	0.55	− 0.66	− 0.34	1.59
AdjIntCov	0.72	− 0.12	0.27	0.91	0.28	0.12	− 0.20	− 0.09
IntCovRatio	0.57	0.11	0.28	0.90	0.28	0.12	− 0.19	− 0.20
5YRel_CACL	0.52	− 0.19	0.83	0.10	0.06	− 0.66	0.62	0.50
IndRel_ChgSalesMargin	− 0.45	1.08	− 0.11	0.67	− 0.10	− 0.18	0.43	0.39
ROA60MSlope	1.90	0.62	− 1.39	0.58	− 0.16	− 1.71	0.99	0.74
ChgSalesMargin	− 0.44	1.00	− 0.36	0.57	− 0.16	− 0.22	0.46	0.50
LTDE	3.07	0.34	− 2.05	0.69	0.80	− 0.09	− 0.12	− 1.36
IndRel_LTDA	1.98	0.38	− 1.06	0.27	0.23	0.12	− 0.23	− 0.46
5YRel_ChgSalesMargin	− 0.62	1.01	0.46	0.10	0.10	0.32	0.18	− 0.34
InvTurn	1.58	0.72	− 0.93	− 0.08	− 0.92	− 0.30	0.89	0.21
5YRel_LTDA	0.61	0.38	0.69	0.40	− 0.84	− 0.12	− 0.47	0.48
WCapToSales	− 1.84	0.77	1.80	− 0.22	− 0.97	0.08	0.35	1.04
IndRel_IntCovRatio	0.53	0.38	− 0.29	0.41	0.57	− 0.05	− 0.53	− 0.07
5YRel_AdjAccruals	0.40	0.41	− 0.38	0.16	0.04	− 0.06	− 0.01	0.27
Chg3YEPS	1.05	1.87	− 2.32	0.70	− 1.46	− 2.67	1.67	1.98
SGPToSales	− 1.80	0.27	1.39	− 0.66	− 0.46	1.08	0.67	0.06
UnexpectedInvChg	0.38	− 0.33	0.36	− 0.15	0.35	0.17	− 0.19	− 0.05
IndRel_LTDE	1.12	0.03	− 1.21	0.69	0.37	0.25	0.12	− 0.84
SalesToEPSChg	− 0.50	− 0.01	1.28	0.20	0.14	0.10	− 0.51	− 0.19
3YAvgAnnEPSGrw	0.97	1.45	− 1.92	0.66	− 1.09	− 2.23	1.53	1.13
DIVIDENDGROWTH	0.08	0.61	− 0.76	0.59	− 0.36	− 0.89	0.73	0.49
InvToAsset	0.04	1.00	− 0.80	0.35	− 1.00	− 1.72	1.02	1.55
EPSEstDispFY2C	0.88	− 0.27	− 0.65	0.04	− 0.23	0.54	0.48	− 0.46
IndRel_QuickRatio	− 1.99	0.38	1.71	− 0.16	− 0.82	− 0.27	0.28	1.18
RONA	1.01	2.17	− 2.45	− 0.31	− 2.06	− 2.91	2.58	2.19
DivperShareGrowth	− 0.09	0.41	− 0.52	0.58	− 0.41	− 0.87	0.65	0.38
AstAdjChg3YEPS	0.98	1.79	− 2.51	0.56	− 1.62	− 2.72	1.62	1.97
NetProfitMargin	0.81	1.88	− 1.31	− 0.62	− 2.17	− 2.49	2.13	1.81
Chg3YCF	1.21	1.67	− 2.18	0.56	− 1.46	− 2.99	1.47	1.72
IndRel_InvToAsset	− 0.16	1.16	− 0.85	0.39	− 0.88	− 1.39	0.98	0.73
IndRel_InvTurn	0.09	− 0.60	− 0.59	0.24	− 0.23	0.14	0.08	0.85
IndRel_EPSEstDispFY1C	− 0.41	− 0.27	0.18	0.30	− 0.39	0.86	− 0.22	− 0.08
5YRel_WCA	0.84	− 0.31	0.08	− 0.08	− 0.03	− 0.60	0.10	− 0.02
GPMargin	− 0.32	1.51	− 0.95	− 1.10	− 1.75	− 0.90	1.95	1.50
IndRel_PAdjChg3YSales	− 0.02	1.05	− 0.66	0.10	− 0.51	− 0.33	1.24	− 0.98
IndRel_SGPToSales	− 1.41	− 0.56	0.90	0.71	0.70	− 0.40	− 1.58	1.51
QuickRatio	− 2.14	0.89	1.48	− 0.51	− 1.76	− 0.61	0.76	1.73
IndRel_SGPToSalesChg1Y	− 0.48	0.66	0.24	0.02	0.54	0.72	− 1.57	− 0.28
Chg3YOPM	1.30	1.22	− 2.24	0.11	− 1.21	− 1.77	1.29	1.13
AstAdjChg3YCF	0.80	1.91	− 2.50	0.75	− 1.91	− 3.18	1.55	2.19
WCapToAst	− 2.36	0.85	1.54	− 0.85	− 1.56	− 0.54	0.79	1.69
5YRel_ROA	0.67	1.14	− 1.27	0.58	− 1.28	− 2.00	0.79	0.81
5YRel_OEA	0.67	1.14	− 1.27	0.58	− 1.28	− 2.00	0.79	0.81
IndRel_CACL	− 2.27	0.37	1.75	− 0.76	− 0.91	0.00	0.29	0.97
WCA	− 2.45	0.82	1.54	− 0.73	− 1.53	− 0.49	0.67	1.58
WCA_2	− 2.48	0.83	1.57	− 0.74	− 1.49	− 0.51	0.63	1.56
5YRel_GPMargin	0.66	0.33	− 1.12	0.36	− 0.72	− 1.16	0.43	0.59
5YRel_WCTurn	− 0.54	0.66	0.32	0.04	− 0.58	− 0.84	0.02	0.29
FinLev	0.74	0.58	− 0.03	0.65	0.23	− 1.79	− 0.54	− 0.52
LogAssets	5.55	1.07	− 5.02	− 0.22	− 0.04	− 1.49	0.39	− 0.93
5YRel_PTMargin	0.93	1.15	− 1.74	0.43	− 1.01	− 1.74	0.73	0.53
5YRel_FCFP	0.38	− 0.39	− 0.56	− 0.26	− 0.32	− 0.47	0.44	0.43
5YRel_OPM	1.13	1.14	− 1.79	0.28	− 1.11	− 1.53	0.74	0.39
Chg1YLTDA	0.35	− 0.10	0.25	− 0.52	− 0.24	− 0.33	− 0.19	− 0.01
IndRel_TotalAccruals	− 0.44	0.06	− 0.24	0.37	− 0.18	− 0.87	− 0.68	1.20
CACL	− 2.53	0.74	1.63	− 0.76	− 1.53	− 0.55	0.72	1.46
CFIC	0.77	1.92	− 2.44	− 0.47	− 2.42	− 3.17	2.60	2.33
EstDiffC	0.13	0.43	− 1.40	0.11	− 1.09	− 0.15	0.02	1.03
EBITMargin	0.80	1.61	− 2.17	− 0.46	− 1.76	− 2.14	1.70	1.49
IndRel_DepToCapex	− 0.55	− 1.24	0.78	0.73	0.89	0.49	− 2.51	0.43
5YRel_EBITMargin	0.98	1.13	− 1.81	0.19	− 1.23	− 1.46	0.75	0.42
IndRel_WCTurn	0.45	0.28	0.01	0.25	− 0.42	− 0.20	− 0.11	− 1.28
ROE	1.64	1.78	− 3.33	− 0.64	− 2.24	− 2.87	2.51	2.10
IndRel_WCA	− 1.53	0.16	0.91	− 0.35	− 1.00	− 0.54	0.36	0.89
CashAst	− 0.68	0.95	0.48	− 0.95	− 2.10	− 1.38	1.45	1.12
AstAdjChg3YFCF	− 0.13	− 0.84	− 0.71	− 0.36	− 0.07	0.13	0.43	0.45
EPSEstDispFY1C	0.09	− 0.59	− 0.04	− 0.11	− 0.61	1.69	− 1.17	− 0.41
DepToCapex	0.29	− 1.48	0.93	− 0.24	1.12	0.96	− 1.25	− 1.50
IndRel_ROE	1.07	1.74	− 1.86	− 1.09	− 2.00	− 2.21	2.00	1.14
IndRel_DebtChg1Y	− 0.28	− 0.34	0.19	0.68	− 0.11	− 1.04	− 0.42	0.07
Chg3YFCF	0.24	0.97	− 0.43	− 1.04	− 0.81	− 0.38	0.58	− 0.41
5YRel_ROE	0.31	1.13	− 1.36	0.56	− 1.36	− 1.97	0.59	0.80
5YRel_ROIC	0.67	0.92	− 1.48	0.31	− 0.98	− 1.81	0.45	0.57
5YRel_CashAst	1.05	− 0.14	− 0.10	− 0.11	− 1.03	− 1.28	− 0.04	0.22
CFAst	0.01	1.85	− 1.99	− 0.58	− 2.60	− 3.15	2.31	2.46
PAdjChg3YFCF	0.00	− 1.10	− 1.13	− 0.06	0.24	− 0.01	0.04	0.29
AdjEPSNumRevFY1C	0.07	0.33	− 1.38	0.04	− 1.54	− 0.21	− 0.02	0.95
IndRel_InvToAst	0.23	0.07	− 0.04	− 0.60	0.08	− 0.37	− 0.42	− 0.79
IndRel_CashAst	− 0.17	0.22	0.41	− 0.87	− 1.38	− 1.66	1.17	0.45
IndRel_ROA	0.07	1.31	− 2.46	− 0.46	− 2.64	− 2.15	2.29	2.18
IndRel_OEA	0.07	1.31	− 2.46	− 0.46	− 2.64	− 2.15	2.29	2.18
CashRatio	− 1.29	0.98	0.97	− 1.07	− 2.28	− 1.37	1.13	1.05
IndRel_NetProfitMargin	0.17	1.30	− 0.33	− 0.72	− 1.95	− 0.26	0.75	− 0.85
AstAdjChg3YOCF	0.10	− 0.08	− 1.79	− 0.20	− 1.04	− 1.38	1.04	1.39
AstAdjChg1YFCF	− 0.14	− 0.70	− 0.66	− 0.56	− 0.14	− 0.14	0.53	− 0.16
IndRel_ROIC	1.22	1.39	− 1.90	− 0.16	− 1.98	− 2.08	0.82	0.72
IndRel_ChgDeprCapex	− 1.27	− 0.25	0.95	0.25	0.40	− 0.63	− 0.98	− 0.57
IndRel_CapAcqRatio	− 0.05	− 0.36	− 0.68	− 0.16	− 1.70	− 0.41	1.28	− 0.09
ROIC	0.29	1.37	− 2.45	− 0.50	− 2.34	− 2.95	2.17	2.22
IndRel_FCFEV	0.98	− 0.27	− 0.82	− 0.82	− 0.40	− 0.92	0.13	− 0.09
IndRel_FCFP	0.76	− 0.20	− 0.91	− 1.13	− 1.58	− 0.62	1.20	0.14
IndRel_GPMargin	− 0.76	0.78	− 1.04	− 0.37	− 1.50	− 1.09	0.70	0.90
ROA	0.10	1.75	− 2.25	− 0.72	− 2.65	− 3.33	2.31	2.38
OEA	0.10	1.75	− 2.25	− 0.72	− 2.65	− 3.33	2.31	2.38
AdjEPSNumRevFY2C	− 0.49	0.63	− 0.21	− 0.37	− 1.69	− 0.21	0.08	− 0.14
PAdjChg3YEPS	0.99	1.23	− 3.02	0.51	− 1.46	− 2.20	0.80	0.74
MVEToTL	− 2.86	0.20	1.57	− 0.75	− 1.67	− 0.58	0.41	1.23
IndRel_WCapToSales	− 1.09	0.46	0.82	0.06	− 0.18	− 1.42	− 1.71	0.60
SolvencyRatio	− 0.95	1.48	− 0.98	− 0.79	− 2.62	− 2.79	1.84	2.25
ROA_2	0.03	1.74	− 2.35	− 0.73	− 2.65	− 3.29	2.25	2.36
AstAdjChg1YOCF	− 0.12	− 0.24	− 1.29	− 0.54	− 0.74	− 0.90	0.96	0.16
IndRel_CapExToAst	− 0.60	0.61	− 1.05	− 0.14	− 1.46	− 2.00	0.81	1.09
IndRel_PAdjChg1YFCF	− 1.55	− 0.98	− 0.08	− 0.03	0.71	− 1.18	0.32	− 0.02
CFEqt	1.54	2.10	− 3.06	− 0.44	− 2.42	− 3.69	1.47	1.63
Chg3YAstTo	0.19	− 0.12	− 1.74	− 0.39	− 0.15	− 0.58	− 0.03	− 0.08
IndRel_PTMargin	0.07	1.25	− 0.52	− 0.79	− 2.21	− 0.75	1.94	− 1.97
IndRel_Chg1YOPM	1.08	1.90	− 1.65	− 0.31	− 1.84	− 2.33	0.58	− 0.43
IndRel_RecTurn	0.44	− 0.45	− 1.19	− 0.33	− 0.92	− 0.60	0.19	− 0.15
AstAdjChg1YCF	0.62	1.06	− 2.97	− 0.04	− 2.06	− 2.47	1.44	1.39
5YRel_Chg1YEPS	− 0.47	0.72	− 0.92	− 0.02	− 0.89	− 1.64	0.02	0.16
EbitToAst_2	0.51	1.48	− 2.99	− 0.53	− 2.25	− 3.25	1.89	2.08
IndRel_EBITMargin	− 0.40	1.67	− 1.00	− 0.77	− 1.86	− 0.71	1.17	− 1.18
IndRel_OPM	0.18	1.24	− 1.09	− 0.73	− 2.00	− 0.61	1.27	− 1.36
Chg1YFCF	0.27	− 0.69	− 0.69	− 0.98	− 1.33	− 0.23	0.96	− 0.40
IndRel_Chg1YOCF	0.00	− 0.29	− 1.23	− 0.84	− 1.26	− 0.73	0.36	0.87
IndRel_PAdjChg1YSales	− 0.16	0.98	− 1.20	− 0.10	− 1.57	− 0.42	− 0.54	− 0.32
EPSNumRevFY1C	− 0.07	− 0.25	− 1.67	− 0.57	− 0.69	− 0.96	− 0.22	1.09
AstAdjChg1YEPS	0.40	0.85	− 2.85	− 0.17	− 2.10	− 2.25	1.36	1.30
SalesToInvCap	0.11	− 0.42	− 1.58	0.11	− 0.50	− 1.53	0.19	0.13
IndRel_PAdjChg1YEPS	0.21	− 0.43	− 1.26	− 0.57	0.21	− 1.32	− 0.37	0.03
PAdjChg3YOCF	0.20	− 0.37	− 2.10	0.01	− 0.40	− 1.06	− 0.13	0.30
FCFEV	0.38	− 0.62	− 0.80	− 0.89	− 1.18	− 1.02	0.30	0.29
NIStab	0.71	1.40	− 2.22	− 0.55	− 2.23	− 2.94	1.16	1.11
Chg3YOCF	0.32	0.21	− 1.71	− 0.80	− 1.62	− 1.94	0.30	1.65
5YRel_RecTurn	− 0.23	0.33	− 0.64	− 0.29	− 0.99	− 1.78	− 0.16	0.09
IndRel_AssetTurn	0.86	− 0.35	− 1.48	− 0.47	− 1.00	− 1.81	0.59	− 0.05
LogTTMSales	4.96	0.65	− 5.35	− 0.44	− 0.90	− 2.49	0.43	− 0.66
IndRel_EPSToSalesChg1Y	− 0.10	− 0.27	− 0.22	− 0.55	− 1.28	− 1.27	− 0.74	0.61
FCFP	0.46	− 0.66	− 0.91	− 1.03	− 1.14	− 1.03	0.33	0.13
Altman_ZScore	− 1.88	0.48	0.33	− 0.99	− 2.48	− 1.86	1.04	1.39
Chg1YGPMargin	0.32	− 0.01	− 1.90	− 0.31	− 1.31	− 1.62	0.45	0.39
Chg1YOPM	0.82	0.91	− 2.40	− 0.29	− 1.77	− 2.83	0.80	0.75
IndRel_Chg1YFCF	0.43	− 0.67	− 0.53	− 0.91	− 1.18	− 0.55	− 0.24	− 0.42
6MChgTgtPrc	− 0.61	0.11	− 1.03	− 0.71	− 0.38	− 0.87	− 1.59	1.00
IndRel_PAdjChg1YCF	− 0.86	0.16	− 0.92	0.14	− 0.86	− 1.74	0.11	− 0.17
IndRel_FCFEqt	0.25	− 0.64	− 0.69	− 1.45	− 1.09	− 1.11	0.36	0.03
IndRel_CFROIC	0.58	0.05	− 2.11	− 1.53	− 1.71	− 2.07	1.64	0.79
Chg1YEPS	0.52	1.30	− 2.77	− 0.33	− 2.72	− 3.31	1.17	0.91
PAdjChg3YCF	0.71	0.82	− 3.41	0.36	− 1.61	− 2.61	0.14	0.31
CapAcqRatio	0.01	− 0.61	− 1.06	− 1.55	− 1.48	− 1.10	0.42	0.08
IndRel_Chg1YEPS	− 0.27	1.57	− 1.55	− 0.26	− 2.12	− 2.83	0.30	− 0.15
FCFEqt	− 0.11	− 0.87	− 0.94	− 1.42	− 1.42	− 1.22	0.52	− 0.09
AssetTurn	− 0.73	− 0.45	− 1.43	− 0.28	− 1.33	− 2.14	0.28	0.51
AssetTurn_2	− 0.75	− 0.45	− 1.45	− 0.27	− 1.38	− 2.06	0.27	0.51
Rev3MFY1C	0.21	− 0.13	− 2.01	− 0.69	− 1.65	− 1.89	− 0.77	1.33
PAdjChg1YEPS	0.38	0.40	− 3.31	− 0.20	− 2.17	− 2.04	0.63	0.68
CashBurn	0.33	− 1.40	− 0.81	− 1.80	− 0.34	− 0.55	− 0.37	− 0.69
PAdjChg3YSales	− 0.24	0.86	− 2.73	− 0.07	− 0.89	− 1.70	− 0.48	− 0.40
IndRel_FwdFCFPC	1.64	− 1.52	− 2.79	− 1.12	− 0.66	− 1.32	0.56	− 0.44
Chg1YROA	0.34	− 0.07	− 2.69	− 0.25	− 1.95	− 2.00	0.72	0.21
6MTTMSalesMom	− 0.43	0.08	− 1.11	− 0.64	− 2.04	− 0.86	0.16	− 0.97
Chg1YCF	0.89	0.91	− 2.81	− 0.05	− 2.61	− 3.72	1.02	0.52
Piotroski_FScore	0.12	− 0.29	− 1.80	− 0.71	− 1.05	− 1.87	0.00	− 0.33
Rev3MFY2C	− 0.59	− 0.20	− 1.85	− 0.48	− 1.08	− 1.73	− 0.54	0.43
IndRel_NCAP	− 0.32	− 1.27	− 0.32	− 1.14	− 0.45	− 0.68	− 0.55	− 1.48
RecTurn	1.03	− 0.40	− 1.75	− 1.07	− 1.23	− 2.25	0.60	− 1.24
BuyToSellRecLess3MSMA	− 0.69	− 0.58	− 1.74	0.34	− 0.25	− 0.97	− 2.35	− 0.12
CFROIC	0.49	− 0.43	− 2.23	− 1.49	− 1.91	− 2.61	1.11	0.65
3MSalesMom	− 0.70	− 0.14	− 0.53	− 1.07	− 1.82	− 0.87	− 0.44	− 0.94
OCFEqt	0.92	− 0.42	− 2.67	− 1.45	− 1.77	− 2.48	0.98	0.35
IndRel_SEV	0.13	− 0.86	− 0.39	− 0.41	− 0.51	− 0.23	− 2.09	− 2.27
AdjRevMagC	0.66	− 0.19	− 3.11	− 0.84	− 1.42	− 2.01	− 0.20	0.46
BuyToSellRecLess3MEMA	− 0.97	− 0.63	− 1.83	0.14	− 0.53	− 0.58	− 2.48	− 0.14
IndRel_CashEV	0.10	− 0.99	− 0.76	− 0.03	− 0.61	− 0.77	− 1.41	− 2.66
ChgATO_2	− 1.20	− 0.52	− 1.93	− 0.33	− 1.27	− 1.07	− 0.22	− 0.75
IndRel_OCFP	0.61	− 1.05	− 2.14	− 1.21	− 1.78	− 1.04	− 0.30	− 0.51
Chg1YAstTo	− 1.12	− 0.58	− 1.90	− 0.42	− 1.22	− 1.45	− 0.16	− 0.61
OCFRatio	− 0.31	− 0.30	− 1.50	− 1.72	− 2.49	− 2.71	0.83	0.73
IndRel_OCFEV	1.01	− 0.75	− 1.47	− 1.24	− 1.07	− 1.17	− 1.22	− 1.64
OCFAst_2	0.22	− 0.43	− 2.28	− 1.66	− 2.42	− 2.95	1.05	0.65
RevMagFY1C	0.83	− 0.19	− 3.15	− 0.92	− 1.51	− 2.51	− 0.58	0.16
IndRel_SP	0.05	− 2.44	− 2.33	− 0.46	1.17	− 1.08	− 1.29	− 1.50
FwdFCFPC	− 0.38	− 1.55	− 2.51	− 0.71	− 0.25	− 0.68	− 0.93	− 1.33
6MChgTgtPrcGap	− 3.67	− 1.61	− 0.45	0.33	− 0.61	0.79	− 2.15	− 1.06
PAdjChg1YSales	− 1.11	0.75	− 2.90	− 0.45	− 2.03	− 1.99	− 0.34	− 0.81
IndRel_CFP	− 0.26	0.45	− 1.94	− 1.94	− 2.92	− 1.07	− 0.32	− 1.34
IndRel_EP	− 0.26	0.81	− 2.76	− 1.23	− 2.36	− 2.00	0.12	− 1.73
IndRel_AstP	− 0.45	− 1.82	− 2.65	− 0.57	1.11	0.10	− 2.83	− 2.33
IndRel_OEP	− 0.38	0.74	− 3.13	− 1.22	− 2.35	− 1.80	0.35	− 1.83
IndRel_CFEV	− 0.17	− 1.03	− 0.98	− 1.69	− 1.88	− 0.94	− 0.84	− 2.22
IndRel_EBITDAP	− 0.52	− 0.67	− 2.75	− 1.21	− 2.41	− 1.07	− 0.41	− 0.75
REToAst	− 0.44	− 0.31	− 1.61	− 1.81	− 3.01	− 3.09	0.49	− 0.04
IndRel_GFP	− 0.11	0.39	− 2.74	− 1.93	− 2.66	− 1.25	− 0.02	− 1.56
IndRel_PTIP	− 0.20	0.58	− 3.45	− 1.49	− 3.03	− 1.32	− 0.19	− 0.79
REToAst_2	− 0.52	− 0.29	− 1.58	− 1.81	− 3.02	− 3.09	0.40	− 0.07
5YRel_OCFP	− 1.48	− 1.35	− 0.75	− 0.93	− 1.91	− 1.69	− 1.45	− 1.28
IndRel_EBITDAEV	0.38	− 1.31	− 1.51	− 1.94	− 2.12	− 1.43	− 1.39	− 2.18
4WChgFwd12MEPS	− 3.20	− 2.51	− 2.28	− 1.57	− 1.81	− 0.50	1.01	− 0.72
CurLiaP	0.75	− 2.89	− 3.21	− 0.86	0.58	0.44	− 2.99	− 3.41
NCAP	− 3.73	− 1.49	0.23	− 1.95	− 1.98	− 0.81	− 1.58	− 0.64
IndRel_BP	− 0.77	− 2.04	− 1.80	− 0.62	− 0.53	− 0.56	− 3.43	− 2.27
IndRel_CashP	− 0.09	− 2.00	− 1.91	− 1.53	− 0.47	− 0.98	− 2.78	− 2.39
IndRel_DivP	− 0.10	− 2.11	− 2.37	− 2.67	− 1.43	− 0.71	− 2.04	− 1.46
8WChgFwd12MEPS	− 5.17	− 2.26	− 1.41	− 1.80	− 0.43	− 1.04	0.05	− 2.95
OCFP	0.26	− 2.24	− 3.71	− 2.03	− 1.77	− 2.27	− 1.73	− 1.88
5YRel_CFEV	− 1.26	− 1.86	− 1.28	− 1.11	− 2.13	− 1.99	− 2.96	− 2.88
AdjEBITP	0.02	− 1.16	− 5.61	− 1.67	− 2.12	− 2.58	− 1.52	− 1.50
OCFEV	− 0.09	− 2.26	− 3.68	− 1.93	− 2.13	− 2.52	− 1.85	− 1.93
OEP	0.03	− 1.25	− 5.62	− 2.29	− 2.92	− 3.45	− 1.03	− 1.21
5YRel_CFP	− 2.49	− 1.93	− 1.00	− 1.44	− 3.05	− 2.35	− 3.00	− 3.14
5YRel_EBITDAP	− 2.76	− 1.32	− 1.84	− 1.45	− 3.42	− 2.64	− 2.79	− 3.01
CashEV	− 1.53	− 2.84	− 2.79	− 2.51	− 2.44	− 1.57	− 2.58	− 2.98
CashP	− 0.95	− 2.82	− 2.95	− 2.57	− 1.98	− 1.46	− 3.07	− 3.46
AstP	− 0.22	− 3.88	− 3.86	− 1.85	− 0.17	− 0.08	− 4.47	− 4.93
GFP	0.08	− 1.28	− 5.80	− 2.35	− 3.18	− 3.56	− 1.99	− 1.65
SP	− 0.78	− 3.67	− 4.30	− 1.74	− 0.92	− 1.39	− 3.89	− 3.86
EBITDAEV	0.21	− 2.54	− 5.72	− 2.22	− 2.58	− 3.23	− 2.60	− 2.67
SEV	− 0.97	− 3.67	− 4.08	− 1.76	− 1.64	− 2.07	− 4.04	− 3.80
BP	− 2.18	− 4.94	− 4.16	− 2.98	− 1.67	− 0.96	− 5.26	− 5.54
5YRel_AstP	− 4.11	− 3.76	− 2.03	− 2.64	− 3.56	− 1.79	− 5.18	− 5.15
5YRel_BP	− 3.68	− 3.57	− 1.81	− 3.10	− 3.90	− 2.23	− 5.23	− 5.40

2. Technical indicators’s Rank IC from 2015 to 2022.

**Table:**

	2015 (%)	2016 (%)	2017 (%)	2018 (%)	2019 (%)	2020 (%)	2021 (%)	2022 (%)	(%)
STO	3.60	5.92	7.84	6.93	5.87	6.12	6.22	7.04	6.19
AnnVol1M	− 0.01	4.91	6.98	5.11	6.37	6.29	8.39	7.94	5.75
5DVolSig	6.65	7.05	4.58	5.58	6.96	3.81	4.51	6.05	5.65
PM1M	6.67	8.72	3.50	6.39	8.08	2.61	2.86	4.82	5.46
PrcTo260DL	8.51	7.02	2.69	4.07	5.23	2.80	6.09	6.93	5.42
IndRel_PM1M	6.53	8.66	3.52	6.06	7.88	3.05	3.20	4.41	5.41
Chg1YTurnover	5.06	6.83	4.32	5.61	5.47	3.69	5.51	6.39	5.36
14DayRSI	8.79	7.68	4.70	4.51	7.11	1.43	1.83	4.31	5.05
10DMACD	9.35	5.85	2.03	4.66	5.00	2.35	4.83	5.98	5.00
IndRel_PM5D	6.38	7.14	3.44	4.06	4.28	2.82	3.55	3.46	4.39
MaxRetPayoff	1.63	4.28	5.88	4.53	4.32	4.02	4.84	5.43	4.37
PM5D	6.27	7.16	3.43	4.19	4.38	2.70	3.05	3.52	4.34
IndRel_50DVolSig	4.69	4.99	2.98	4.57	3.49	3.66	4.92	4.86	4.27
PM6M	10.11	6.06	− 0.33	2.91	3.60	0.89	4.46	6.34	4.25
50DVolSig	4.56	4.68	3.02	4.31	3.66	3.25	5.27	5.22	4.24
AnnVol12M	− 1.69	3.83	5.81	4.02	3.92	5.45	5.62	6.08	4.13
IndRel_PM6M	10.13	5.83	− 0.43	2.60	3.46	1.14	4.24	5.63	4.08
AdjSTO_6M	0.48	3.27	6.16	5.06	4.31	4.89	3.66	4.39	4.03
RSI26W	9.40	5.32	− 0.52	2.70	3.15	0.30	4.02	5.62	3.75
IndRel_MaxRetPayoff	1.49	3.13	4.71	3.43	3.33	3.28	4.66	4.27	3.54
PM9M	8.23	5.56	− 0.54	1.87	3.42	0.65	4.12	4.25	3.44
IndRel_PM9M	8.49	5.63	− 0.59	1.64	3.03	0.59	4.32	3.59	3.34
24MResRtnVar	− 0.66	3.59	3.94	3.13	2.59	3.92	4.36	4.61	3.18
90DCV	− 0.11	2.83	2.84	0.74	4.86	4.61	4.64	4.05	3.06
PM-style	2.85	4.33	3.24	4.15	4.68	2.15	0.32	2.61	3.04
Alpha60M	4.55	3.51	0.40	1.69	2.79	1.82	2.90	4.67	2.79
20DStochastic	5.08	5.26	1.06	3.00	4.31	− 0.79	1.18	2.50	2.70
PA52WL20DLag	4.15	3.41	1.07	2.09	0.75	0.98	4.65	4.08	2.65
5DMoneyFlowVol	5.43	3.79	1.99	1.25	2.85	1.23	1.85	2.46	2.61
SharpeRatio	3.12	5.20	1.45	2.71	0.43	− 0.01	4.72	3.17	2.60
PSlopeSERR_26W	5.72	1.88	− 0.17	2.22	0.90	− 0.65	3.44	4.90	2.28
LogUnadjPrice	3.16	4.21	1.37	1.39	− 1.03	− 1.40	5.14	5.25	2.26
50To200PrcRatio	6.61	2.70	− 1.43	0.36	0.82	− 0.20	3.28	3.41	1.94
StdErr180D	5.73	2.15	− 1.35	1.73	0.46	− 0.96	2.51	3.72	1.75
PrcTo52WH	8.40	3.69	− 2.26	1.47	1.20	− 2.56	1.81	1.90	1.71
PRatio15To36W	5.68	1.86	− 1.73	0.26	0.42	− 0.39	2.57	3.74	1.55
39WRtnLag4W	4.58	2.58	− 1.38	− 0.12	0.14	− 0.73	2.47	2.73	1.28
IndRel_PM12M1M	5.29	1.90	− 1.88	0.02	− 0.17	− 1.39	3.04	0.79	0.95
PM12M1M	4.84	2.09	− 1.70	0.26	− 0.35	− 1.36	2.87	0.71	0.92
52WSlope	3.00	2.78	− 2.06	− 0.96	− 0.70	− 0.63	2.49	1.17	0.63
BookLev	2.94	− 0.16	− 1.61	0.70	1.57	0.46	− 0.26	− 1.27	0.30
Alpha18M6MPChg	0.63	3.59	− 0.48	− 1.19	− 0.81	− 0.47	0.16	0.06	0.19
YoYChgDA	− 0.05	− 0.12	0.09	0.40	0.17	0.35	− 0.07	− 0.03	0.09
Alpha12M6MPChg	1.80	0.90	− 0.64	− 1.90	− 0.13	− 0.16	− 0.57	− 0.65	− 0.17
Beta60M	− 1.15	0.15	1.23	− 0.36	− 0.40	− 1.42	0.23	0.23	− 0.18
VolAdjRtn12M	2.56	0.07	− 3.03	0.10	− 2.44	− 2.25	2.34	0.04	− 0.33
RelPrStr_12M	2.77	0.19	− 3.15	− 0.13	− 2.36	− 2.42	2.45	0.02	− 0.33
Alpha36M6MPChg	0.80	0.24	− 0.76	− 1.87	− 1.13	− 1.23	0.34	0.66	− 0.37
CVVolPrc60D	0.95	− 0.92	0.54	1.56	− 0.62	− 1.58	− 3.39	− 2.84	− 0.79
CVVolPrc30D	0.99	− 0.68	− 0.15	2.60	− 0.19	− 1.59	− 4.54	− 2.74	− 0.79
CVVolPrc20D	1.42	− 0.18	− 0.59	2.05	0.05	− 1.87	− 4.61	− 3.35	− 0.89
STO_6M	− 0.56	− 2.09	− 0.15	0.17	− 0.83	− 0.57	− 1.69	− 2.14	− 0.98
RskAdjRS	− 4.63	0.25	− 1.31	0.13	− 1.47	− 2.56	− 1.52	− 4.62	− 1.97
130DMinRtn	2.02	− 1.39	− 5.38	− 2.08	− 2.01	− 1.81	− 4.14	− 3.00	− 2.22
HL1M	− 3.27	− 4.72	− 1.44	− 3.22	− 4.42	0.67	− 1.07	− 2.52	− 2.50
Chg1YAmihud	− 3.07	− 1.49	− 1.90	− 4.99	− 2.04	− 1.49	− 3.64	− 5.22	− 2.98
4To52WPrcOsc	− 9.12	− 4.36	1.02	− 1.22	− 2.16	− 0.01	− 3.98	− 4.41	− 3.03
HL52W	− 7.09	− 5.07	− 1.11	− 4.11	− 3.16	0.62	− 4.02	− 4.83	− 3.60
Amihud	− 5.14	− 7.18	0.34	− 4.52	− 1.58	− 0.83	− 5.73	− 5.15	− 3.72
Vol-style	1.40	− 3.87	− 5.96	− 3.86	− 5.28	− 5.47	− 6.98	− 7.08	− 4.64
